# The Role of the Lateral Habenula in Suicide: A Call for Further Exploration

**DOI:** 10.3389/fnbeh.2022.812952

**Published:** 2022-03-14

**Authors:** Rocky B. Marks, Janelle Y. Wee, Samantha V. Jacobson, Kimi Hashimoto, Katherine L. O’Connell, Sam Adler Golden, Phillip Michael Baker, Keyne Catherine Law

**Affiliations:** ^1^Department of Clinical Psychology, Seattle Pacific University, Seattle, WA, United States; ^2^Department of Biological Structure, University of Washington, Seattle, WA, United States; ^3^Department of Psychology, Seattle Pacific University, Seattle, WA, United States

**Keywords:** suicide, habenula, lateral habenula, habenula, neurobiomarkers, ideation, capability

## Abstract

Despite decades of significant effort in research, policy, and prevention, suicide rates have continued to rise to the current peak of 14.6 per 100,000 deaths. This has resulted in a concerted effort to identify biomarkers associated with suicidal behavior in the brain, to provide predictions that are better than the chance of discerning who will die by suicide. We propose that the lateral habenula (LHb), and its dysfunction during a suicidal crisis, is a critical component of the transition from suicidal ideations to self-harm. Moreover, the LHb—a key functional node in brain reward circuitry—has not been ascribed a contributory role in suicidal behavior. We argue that the LHb anchors a “suicide circuit” and call for suicide researchers to directly examine the role of the LHb, and its long-term modulation, in response to the negative affect in suicidal behavior. Discerning the neural mechanisms of this contribution will require the collaboration of neuroscientists and psychologists. Consequently, we highlight and discuss research on LHb as it relates to suicidal ideation, suicidal behavior, or death by suicide. In so doing we hope to address the bench-to-bedside translational issues currently involved in suicide research and suggest a developmental framework that focuses on specific structures motivated by theoretical anchors as a way to incorporate neurobiological findings within the context of clinical theory.

## Introduction

Out of every 100,000 deaths that occur in the United States, 14.6 result from self-inflicted injuries (CDC WISQARS, 2020). Suicide rates continue to rise yearly, despite significant research, policy, and prevention efforts across commercial, academic, and government sectors. Five decades of suicide research have not improved the ability to predict who will die by suicide (Franklin et al., 2017), or what interventions effectively prevent it (CDC WISQARS, 2020). Currently, leading theories of suicide risk are based on psychosocial models encompassing the complex interplay between environmental, affective, behavioral, and neurobiological factors.

While acknowledging the role of neurobiology, these theories often reduce the exact functions, structures, and circuitry to an abstract, conceptual influence rather than a tractable avenue for intervention. For example, suicide is thought to be moderately heritable, with the family history of suicide emerging as a salient risk factor for suicidal behavior, especially among families with high rates of psychopathology (Coon et al., 2020). However, the specific genes, the brain structures under their genetic influence, or the neurotransmitters affected by the inherited risk of suicide are much less clear. Conversely, biological research on suicide has largely focused on examining independent mechanisms [e.g., 5-hydroxytryptamine receptors (5-HT), Brain-Derived Neurotropic Factor (BDNF), Hypothalamic-Pituitary-Adrenal (HPA) axis reactivity] that carry the risk of failing to incorporate the dynamic and complex biopsychosocial systems involving suicidality and an individual’s environment. Noting this recurrent pattern of academic conceptual isolationism, we call for increased interdisciplinary investigation into neurobiological correlates of suicidality guided by psychosocial suicide research theory. We provide a candidate for this approach, the lateral habenula, a neural structure that we believe will justify this argument through the lens of suicidology. The pairing of neurobiological mechanisms with suicide theory, long overdue, has the potential to dramatically improve clinical practice.

## Psychosocial Suicide Theories and Research—A Modern Day Primer

Many psychosocial theories propose explanations for why people die by suicide and identify factors that may contribute to the transition of suicidal ideation into suicidal behavior. The Stress–Diathesis Model of suicide (van Heeringen, 2012) proposes that stressors such as psychological disorders, crises, poverty, hopelessness, and adverse childhood experiences interact with epigenetic and neurobiological alterations to increase suicide risk. However, not all individuals who experience stressors think about suicide or engage in suicidal behavior, suggesting that neurobiological and genomic vulnerabilities contribute to the development of suicide risk, and thus a stress–diathesis interaction occurs. In support of this theory, structural and functional brain states can serve a role in distinguishing who, from samples of persons who experienced similar painful life events, will exhibit thoughts of suicide (Mann and Rizk, 2020). Further, the Stress–Diathesis Model prominently uses the concept of the “suicidal mode”, which defines suicide-specific motivations, cognitions, and affect in the context of the stressors which elicit them. With each activation of the suicidal mode, suicidal behavior becomes progressively more accessible in memory and the suicidal mode is more responsive to minor stimuli. With repeated stimulation, the suicidal mode becomes activated more easily and with a lower threshold for activation (Leon et al., 1990; Oquendo et al., 2004). It stands to reason that the facilitation of the suicidal mode is accompanied by changes in neural plasticity in key brain regions associated with processing these stimuli but, as of the time of this writing, these studies have yet to be done.

Additional theories explain the progress of suicidal ideation to lethal self-injury. The Interpersonal Theory of Suicide (IPTS; Joiner, 2005) states that an individual: (i) must feel as if they are a burden on others; and (ii) feel as if they are disconnected from their social environment, to begin thinking of killing themselves. Additionally, the IPTS specifies that to act on suicidal ideation, an individual must acquire the capability for suicide, which involves an increased pain tolerance and fearlessness of death that allows them to overcome the basic drive towards self-preservation inherent in biological life. In support of this theory, past studies have found that an acquired capability for suicide, the ability to tolerate pain, and fearlessness about death differentiates those who think about suicide from those who make a suicide attempt (May and Klonsky, 2016; Paashaus et al., 2019). It is hypothesized that acquired capability was developed from repeated exposure to painful and provocative experiences (e.g., non-suicidal self-injury; Bender et al., 2012; Bauer et al., 2020). Building on the IPTS, the Three-Step Theory (3ST; Klonsky and May, 2015), incorporates that pain and hopelessness, together, initiate passive suicidal ideation (“I do not want to exist”) that is escalated into active suicidal ideation (“I want to kill myself”) when there is a lack of connection with others. The 3ST also extends acquired capability for suicide to include practical capability (having knowledge/access to the means for suicide) and dispositional capability (innate neurobiological/genetic factors that enable an individual to enact lethal suicidal behavior). Here again, it is possible that brain regions associated with pain and fear modulation contributes to changes in suicide capability. Confirmation of this would help solidify these theories and provide potential biomarkers that can contribute to the progression from suicidal ideation to suicidal behavior.

Integrating psychosocial theories, the Integrated Motivational Volitional Theory (IMVT; O’Connor and Kirtley, 2018), conceptualizes the progression of suicide into three phases. First is the pre-motivational phase, which includes a diathesis–stress approach that promotes the development of suicidal ideation. Next, the motivational phase leads to the expression of suicidal ideation that emerges as a response to feelings of entrapment, hopelessness, and defeat. In the final volitional phase, acquired capability, exposure to others’ suicidal behaviors, detailed suicide plans, and cognitive rehearsal of death and suicide facilitates the transition from suicidal ideation into suicidal behavior.

Other theories have integrated the idea of suicidal mode that explain the fluidity of factors that trigger and contribute to the severity and duration of an individual’s suicidal state. When internal or external precipitants trigger the suicidal mode, it acts in a synchronistical fashion to produce a suicidal episode characterized by cognitive themes, physiological arousal, and death-related behaviors. Extending the idea of suicide risk as dynamic, as opposed to stable, is the Fluid Vulnerability Theory (FVT; Rudd, 2006). This theory conceptualizes suicide risk as a combination of baseline risk factors (e.g., past suicide attempts, depressive symptoms, etc.) and acute risk factors (e.g., sudden losses, major life changes, etc.). Based on the conceptualization of dynamic suicide risk, the National Institute of Mental Health has established a priority initiative to examine time-varying suicide risk factors (Kleiman et al., 2017; Hallensleben et al., 2018; Bryan et al., 2019; NIMH, 2020).

Psychosocial research on suicide contains a diverse range of viewpoints, ranging from those emphasizing the physical, such as pain tolerance (Rabasco and Andover, 2020), to the implied, such as cultural and economic diversity and social belongingness (Chu et al., 2010; Li Z. et al., 2011). Whether genetic and neurostructural influences are defined as diatheses, dispositional capability, or biological vulnerability to suicidal behavior, theories of suicide implicitly emphasize the role of genetics and biology in determining risk. These theories, however, do not define structures or biomarkers that influence suicidality, and in doing so settle for defining the biology of suicide merely through conceptual abstractionism. Furthermore, absent neurobiological candidates, the foundational ideas of psychosocial suicide risk factors are unable to discriminate between those who will develop suicidal ideation, those who will die by suicide, and those who will never think of lethal self-injury at all (Franklin et al., 2017; Millner et al., 2020). The study of suicide must evolve and use integrative systems approaches that more closely approximate the complex picture of suicidality by incorporating ecological, psychosocial, and neurobiological factors leading to the emergence of suicidal behavior.

### Neurobiological Trends in Suicidology

There are several promising neurobiological targets that contribute to suicidal behavior, revealed in large part by the increasing popularity of identifying suicide-related biomarkers, that hold the potential to identify the most prominent systems or biomarkers implicated in the neuropathology of suicide (Sudol and Mann, 2017; Calati et al., 2020). One of the earliest targets was the serotonergic system, leading to the identification of suicide-associated genetic variations, receptor activity, and downregulation of the neurochemical itself (Stockmeier et al., 1998; Mann, 1999, 2003; Arango et al., 2001; Oquendo et al., 2003, 2006). However, serotonergic suicide-related data has not been consistent across suicide research studies. For example, low midbrain serotonin transporter bindings have been found among suicide attempters (Miller et al., 2013) while greater serotonin receptor binding potential was found in the brainstem of suicide attempters in other studies (Sullivan et al., 2015). Given the inconsistencies in the serotonergic systems’ importance in the neuropathology of suicide, research has extended to examining other neural structures and systems’ role in suicide.

While biological research has progressed our understanding of suicide, conversely neurobiological research can run into difficulties taking mechanistically complex processes and translating them into cognitions or behaviors that suicidal persons may struggle with. In addition, neurobiological research can run the risk of describing specific mechanisms of suicidality that simply need more holistic psychosocial factors to create research with high explanatory power. We argue that, by integrating findings from psychosocial studies of suicide, we can better map our research onto the complex systems of suicidality. To that end, we would call for further examination into the LHb, a structure we believe may be key in developing dynamic suicide research.

## The Lateral Habenula, Suicidal Ideation, Suicidal Behavior, and Capability for Suicide

The LHb is often ignored in neurostructural research due to the technical challenge of resolving its structure because of its small size and deep position within the brain. However, advances in both the power of, and analytical approaches to, resolving fMRI signaling now provide satisfactory resolution for LHb registration and segmentation (Gan et al., 2019; Hashikawa et al., 2020).

Anatomically, the habenula is an epithalamic structure often subdivided into the medial habenula and the LHb linking forebrain and midbrain regions, connecting a host of neural structures that include the ventral pallidum, the ventromedial prefrontal cortex (vmPFC), the lateral hypothalamic area (LHA), the globus pallidus, the dorsal raphe nuclei (DRN) and the ventral tegmental area (VTA; Aizawa and Zhu, 2019; Qiao et al., 2020). The LHb is largely innervated from the entopeduncular nucleus and the lateral hypothalamic area, serving a function as a co-releaser of glutamate and GABA. Indeed, within the LHb, glutamatergic neurons project to the ventral tegmental area and the GABAergic rostromedial tegmental nucleus (Shabel et al., 2012). As a relay interface between basal ganglia and limbic system, the LHb is involved in motivational and emotional control of behavior, playing a critical role in both the stress response and behavioral responses induced by expected reward. Further, the LHb operates as a hub between a host of other neural structures that transforms motivational representation into appropriate behavioral outputs by regulating monoaminergic neurotransmission (Stamatakis et al., 2013; Li J. et al., 2017; Ambrosi et al., 2019). Our rationale for implicating the LHb in suicidality is founded on a broad base of past research which has associated LHb dysfunction with many different concepts closely linked with suicidality.

### The LHb in the Desire and Drive for Suicide

Within the world of suicide research, an emerging therapeutic focus has reconceptualized suicidality on a behavioral continuum. That is to say of the many suicide risk factors established over decades of research, some appear to directly drive suicide-specific thoughts, feelings, and behaviors whereas other risk factors can be thought of as indirect, in that they are circumstances, states, or conditions leading the patient to consider their life not worth living (Tucker et al., 2015; Jobes et al., 2018). The distinction is subtle, yet important, playing into the larger conversation of whether a risk factor largely will influence a person through soliciting thoughts of suicide, or whether a factor may motivate suicidal behavior. In linking the function of the LHb to suicidality, we believe it is helpful to contextualize past research on this continuum.

#### The LHb and Desire for Suicide

LHb thought to be a downstream structure in the pathway through which sensory information, projected from other neural structures (such as the medial septum), generates sensory-evoked aversion through a net effect of negatively valenced emotions in animal models (Zhang et al., 2018). This pathway, activated through glutamatergic and GABAergic neurons, may be underlying the process through which the LHb increases the processing of negative stimuli and decreases the processing of positive stimuli (Belzung et al., 2015). This contributes to a neurobiological sensitivity to predict punishment even from neutral stimuli and this process may underlie the pervasive negative emotional state that characterizes depression. Over time, hyperactivity in this pathway’s functional connectivity may contribute to chronic dysregulation of the habenula, leading to long-term alterations of dopamine, serotonin, and norepinephrine transmission that primes a neurological disposition towards avoidance and facilitates social withdrawal related to suicidal ideation (Knowland and Lim, 2018; Ambrosi et al., 2019). In addition, LHb activity has been linked to insomnia, deficits in circadian rhythm regulation, and somatization which are all high arousal life stressors linked with suicidal ideation (Fakhoury, 2017; Mendoza, 2017; Gold and Kadriu, 2019; Qiao et al., 2020).

Further, due to its function as a communication center between limbic structures in the forebrain and aminergic centers in the midbrain, and the structure’s key role in monoamine transmission and cognition, the LHb is closely linked to both the emergence and maintenance of treatment-resistant depressive symptoms, a condition found to co-occur heavily with hopelessness (Papakostas et al., 2005; Lecourtier and Kelly, 2007; Browne et al., 2018). Hopelessness is thought to be a key factor in differentiating suicidal ideators from those who do not experience suicidal thoughts (Wolfe et al., 2019). Indeed, the LHb has been implicated in anhedonia (Li et al., 2013), learned helplessness (Li B. et al., 2011), and rumination (Belzung et al., 2015) which have all been linked to hopeless depression and suicidality in past literature (Abramson et al., 2000). These deficits can be linked to the social disconnectedness underlying the process through which a nonsuicidal person can begin to develop suicidal ideation.

#### The LHb and Drive for Suicide

Hyperactivity in the LHb has been found to be associated with depressive symptomology, anti-reward signaling (systems that activate to limit or diminish rewarding behavior), and impairments in reward-seeking behaviors (Matsumoto and Hikosaka, 2007, 2009; Hong et al., 2011; Proulx et al., 2014).

A further specification of the effect of monoamine theories of suicide relates to the endocannabinoid (eCB) system in the LHb. The eCB system consists of receptors, receptor agonists, and classified as lipid mediators and receptors are typically situated in the presynaptic terminal of the neuron, meaning they are synthesized in an activity-dependent manner compared to neurotransmitters (Shepard and Nugent, 2021). Dysregulation of the eCB system has been implicated in neuropsychiatric conditions, including depression and other stress-related disorders (Castillo et al., 2012; Berger et al., 2018). Endocannabinoids are widely prevalent throughout the brain, suggesting that eCBs are fundamental in modulating synaptic functioning. The eCB system interacts with other neuromodulatory systems and may thus represent an extension of the monoamine theory of depression, as it regulates the release of glutamate, GABA, and other monoamines implicated in depression (Castillo et al., 2012). Notably in the LHb, eCB signaling plays a role in controlling synaptic plasticity, neuronal activity, and subsequent associated behavior, therefore alterations in eCB regulation of LHb neurons contribute to LHb dysfunction that is associated with motivational and social deficits seen in depression and stress-related disorders (Shepard and Nugent, 2021). In the Fluid Vulnerability Theory of suicide, eCB levels in the LHb could then play a crucial role in understanding biological predispositions to suicide risk, which then could be compounded by chronic stress (Authement et al., 2018). The LHb is a critical region that controls the expression of aversive behavior that is activated by aversive stimuli or lack of expected reward.

There is growing evidence that implicates the habenula in stress-related behavioral alterations and identifies endocannabinoids’ role in these processes (Berger et al., 2018; Vickstrom et al., 2021). Stress engages eCB signaling in the LHb, evident by rat exposure to social defeat stress demonstrating increased levels of endocannabinoids and agonist binding affinity in the LHb compared to non-stressed rats (Berger et al., 2018). Similarly chronic stress, linked with emotional dysregulation, affects synaptic transmission and transcriptional plasticity in the LHb. Thus, we propose that emotional dysregulation induced by acute suicide risk factors may result in a sensitization of the LHb, leading to increased LHb activity that is observed during chronic stress (Cerniauskas et al., 2019). Stress may be affecting the development of suicidal ideation through activation of the LHb and subsequent activation of Brown Adipose Tissue (BAT) thermogenesis, in a process called “emotional hyperthermia.” This involves hyperactivity of the LHb and a corresponding subtle increase in body temperature that has been shown to correlate with increases in perseverative and ruminative behaviors frequently associated with suicidal ideation (Law and Tucker, 2018; Brizuela et al., 2019). Maladaptive responses to chronic stress play an important role in the emergence of mood disorders, especially those with biological vulnerability or predisposition. Chronic stress creates a marked increase in LHb activity and increased LHb activity promotes depression-related behavior (Chen and Kenny, 2018; Cerniauskas et al., 2019). As a strong link exists between chronic stress, depression-related thinking, and suicidal ideation (Park et al., 2010), we propose that these biological mechanisms may be a driving force behind processes wherein an individual both begins to desire dying by suicide and further still behind subsequent processes through which an individual is motivated to engage in suicidal behavior.

#### The LHb and Capability for Suicide

Another link to LHb function and suicidality could be through the interpretation of capability for suicide, a term for deficits in pain and fear processing allowing one to overcome the drive towards survival inherent in all biological life. Activity in the LHb is thought to underscore the interpretation of both physiological and psychological pain as aversive stimuli (Wirtshafter et al., 1994; Li Y. et al., 2017), potentially implicating this structure in the induction of feelings of entrapment necessary for one to act on their suicidal thoughts. Indeed, a growing body of literature has indicated that hyperactivity in the LHb may result in both a “reward deficit syndrome,” characterized clinically by anhedonia, and reduced motivation, along with an “enhanced anti-reward syndrome” noticeable as dysphoria, which may be driving suicidal behaviors through entrapment (Qiao et al., 2020). Further, chronic dysregulation of the habenula may lead to long-term alterations of dopamine, serotonin, and norepinephrine transmission that may facilitate social withdrawal maladaptive coping related to entrapment and thus, suicide-related behaviors (Knowland and Lim, 2018; Ambrosi et al., 2019).

Consistent with cytokine theories of depression (Jeon and Kim, 2016; Shariq et al., 2018) activation or neuroplasticity in the LHb may be associated with neuroinflammation found to be related to greater intensity of suicidal ideation (Brundin et al., 2015) and greater risk of a completed suicide attempt (Batty et al., 2016). Rodent models of depression (Zhao et al., 2018; Guan et al., 2020) have found similar increases in proinflammatory factors and proteins in the LHb, including tumor necrosis factor-a (TNF-a), C-reactive protein (CRP), and pro-inflammatory cytokines such as interleukin-1b (IL-1b), IL-6, and IL-10. This increase in neuroinflammation within the LHb appears to be associated with greater depressive symptoms and reductions in fear processing (Zhao et al., 2018), particularly notable for suicidality given the LHb’s involvement in emotion regulation and processing of aversive stimuli (Ambrosi et al., 2019).

The LHb may be involved in modulating fear and pain which are theorized to be core components of suicide capability (Joiner, 2005). The LHb contains a number of pain-activated neurons (PANs), which increase their firing rates when activated by noxious stimulation. As these neurons are activated, the pain threshold decreases, meaning that pain tolerance is reduced and stimuli are more likely to be perceived as painful (Li et al., 2016). Individuals with high pain tolerance, and therefore higher levels of capability for suicide, may experience sensitization in the firing rates of these LHb PANs. Further, this argument suggests that hyperactivity in the LHb may subsequently lead to desensitization to critical cognitive and affective signals such as gratification, danger assessment, and pain sensitivity as shown in past research (Li Y. et al., 2017; Yang et al., 2018). Indeed, a pathway has emerged through with pain signals are received from the spinal cord, leading to PAN activation in the habenula. Over time, chronic stimulation leads to an increase in the excitability of the LHb neurons which suppresses reward responses projected to the VTA (dopaminergic) and DRN (serotonergic), implicating the LHb in both the pain/depression comorbidity (Zhang et al., 2020). Along this pathway, synaptic changes in the LHb resulting from desensitization of these structures would indeed result in the increases in pain tolerance and decreases in pain sensitivity found to consistently co-occur with the painful and provocative events comprising an individual’s acquired capability for suicide (Franklin et al., 2011; Koenig et al., 2016).

Additionally, LHb activity has been implicated in the processing of fear, meaning there is a conceptual link between activation in LHb glutamatergic neurons and increases in fearlessness, particularly in the fearlessness of death (Ribeiro et al., 2014). Results from rodent model research have found that inactivation in the LHb influenced contextual memory deficits and enhanced the fear response to a conditioned stimulus (Durieux et al., 2020). Thus, we believe it is likely that hyperactivity in the LHb could lead to deficits in cueing the fear response in response to suicide-related stimuli. That is, LHb activation may be differentiating between individuals who are high in capability for suicide and those who are low by influencing the likelihood that an individual will perceive suicide-related stimuli as aversive. However past research has found a less robust connection between the habituation to self-injurious or suicidal behavior and resultant increases in capability for suicide (Ribeiro et al., 2020), and thus future research should consider examining whether this hyperreactivity is either static for each person or dynamic and responsive to an individual’s environment.

## Conclusions: Where Do We Go from Here

Consistent with the associations of the LHb with the anti-reward circuit, depressive symptoms, anhedonia, reductions in appetitive behaviors such as socialization, and deficits in stress-related fear responses, we believe the LHb is likely a critical structure for investigations into the neuropathophysiological correlates of suicidality. Based on existing research, there is strong evidence for the habenula’s involvement in suicide and suicide-related behaviors. Specifically, it is plausible that LHb dysfunction may play a critical role in facilitating the transition from suicidal ideation to suicidal behavior.

**Figure 1:**
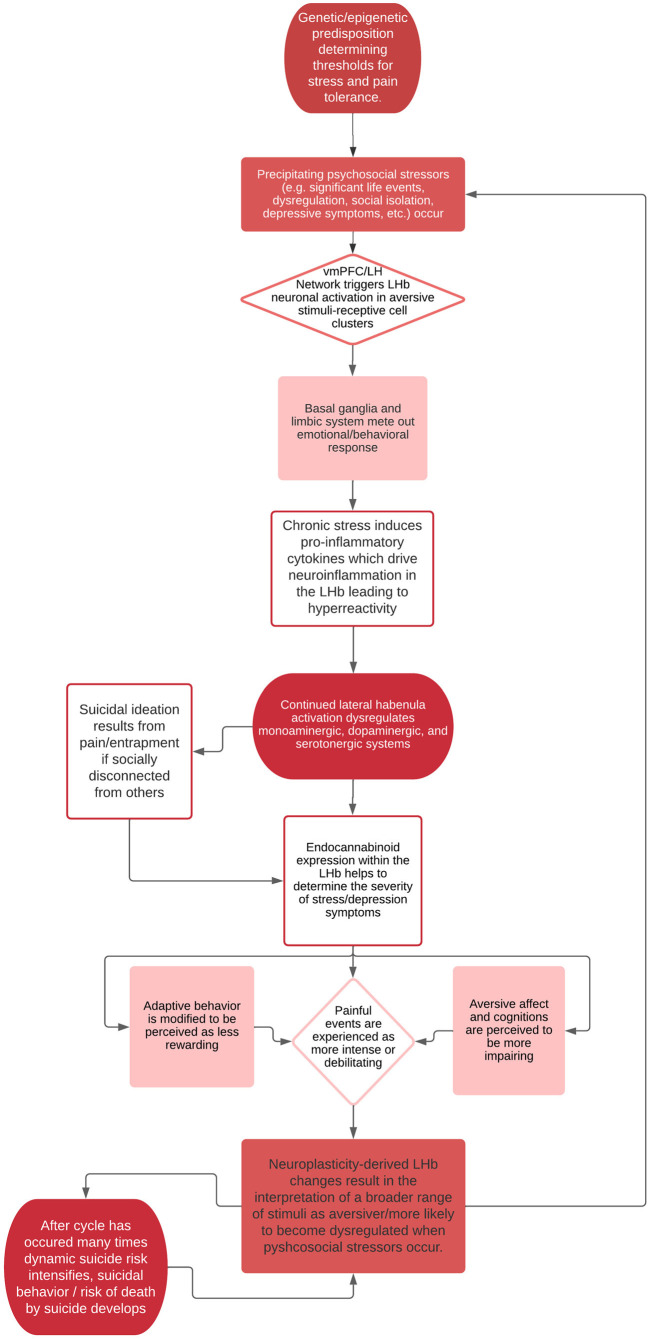
Theoretical process of habenular “suicide pathway”.

In the vein of suicide theory, we believe hyperactivity in the LHb can likely be linked to one’s tendency to perceive events as painful and provocative, key experiences in the IPTS and 3ST theories of suicide. Further, neuroplasticity in the LHb likely plays a crucial role in influencing one’s baseline levels of suicide risk, such that when psychosocial stressors activate acute vulnerabilities for suicide risk (per the IMVT), an individual’s ability to recover and remit from suicidal crises may depend, in part, on neurobiological factors in the LHb (e.g., endocannabinoid receptor density, cytokine levels, and monoaminergic signaling). Once activated, the LHb may sustain negative emotional states through key structures in the basal ganglia and limbic system, promoting an anti-reward strategy that leads to vulnerabilities in suicidal thoughts and behaviors. Chronic activation of the LHb may then result in shifts towards hyperactivity in neuronal cell clusters responsive to aversive stimuli, functioning as a byproduct of the brain’s reaction to dysregulated emotional states linked to psychosocial or environmental cues. This process would then result in a one-way “suicide circuit,” by which the LHb becomes sensitized to aversive stimuli, making the suicidal mode easier and easier to access with each activation. See Figure 1 for a hypothesis of this proposed pathway. As such, the role of the LHb, and its long-term modulation in response to negative stimuli in suicidal behavior should be examined. Given the technical and methodological challenges of studying the habenula and the limitations of making conclusions from animal models to human models, our proposed model is developed based on research’s current understanding of the habenula’s role in various cognitive, affective, and behavioral processes. Thus, our model may be updated as our understanding of the habenula and related circuitry improves.

We believe that a synergistic partnership between interdisciplinary collaborators can yield potentially life-saving research that can be used to optimize suicide risk assessment and prevention practices. Specifically, it would be helpful to use existing suicide theories generated by psychological research to inform neuroscience research using human models and generate hypotheses that can then be tested in animal models. The findings from neuroscience research can then be used to refine suicide theories and applied to optimize suicide risk assessment and prevention practices. As such, we hope that suicide research moving forward will become more interdisciplinary and see more collaboration between neuroscientists and psychologists.

## Author Contributions

RM, JW, and KL contributed to the conception and design of this review. RM, JW, SJ, KH, and KO’C gathered and synthesized review articles and wrote sections of the manuscript. RM organized the database and wrote the first draft of the manuscript, which SG, PB, and KL provided edits and areas of focus. All authors contributed to the article and approved the submitted version.

## Conflict of Interest

The authors declare that the research was conducted in the absence of any commercial or financial relationships that could be construed as a potential conflict of interest.

## Publisher’s Note

All claims expressed in this article are solely those of the authors and do not necessarily represent those of their affiliated organizations, or those of the publisher, the editors and the reviewers. Any product that may be evaluated in this article, or claim that may be made by its manufacturer, is not guaranteed or endorsed by the publisher.
